# Immune Checkpoint Inhibitors and Liver Transplantation: Case Report and Review of the Literature

**DOI:** 10.1002/ccr3.70412

**Published:** 2025-04-14

**Authors:** Iva Skocilic, Alojzije Kosic, Ivona Badovinac, Tajana Filipec Kanizaj, Renata Dobrila Dintinjana, Maja Kolak, Anamarija Bukovica, Sanja Ropac, Ivana Mikolasevic

**Affiliations:** ^1^ Clinical Hospital Center Rijeka Rijeka Croatia; ^2^ Merkur Clinical Hospital Zagreb Croatia; ^3^ Tumor Clinic, Clinical Hospital Center Rijeka Rijeka Croatia

**Keywords:** atezolizumab, breast cancer, immune checkpoint inhibitors, liver transplantation, malignancy

## Abstract

As immunotherapy is becoming more inevitable in everyday oncology, we are witnessing a higher number of patients who are on immunosuppressive medication but are also candidates for immune checkpoint inhibitors (ICIs) treatment. There have been few case reports and several small retrospective studies investigating the use of ICIs in liver transplant recipients mainly with hepatocellular carcinoma and skin cancer, but there is no report regarding the use of atezolizumab for the treatment of metastatic breast cancer in liver transplant recipients. We are presenting a metastatic breast cancer female patient undergoing both immunosuppressive treatment after liver transplantation due to cryptogenic liver failure and anti‐programmed death ligand 1 (PDL1) medication—atezolizumab whose liver enzymes and tacrolimus level we have monitored intensively through 18 months and is still ongoing. Our patient has not presented with any signs of acute graft rejection and has a regression on follow‐up imaging despite the treatment combination.

**Summary**

- The use of immune checkpoint inhibitors (ICIs) treatment following liver transplantation is more challenging; thus, a case‐by case‐based approach is needed in the context of ICI administration in this population of patients.
- Ultimately, a multidisciplinary approach should be a standard of care for each of these challenging patients.

## 1 Introduction

Orthotopic liver transplantation (OLT) is the only potential curative method for end‐stage liver disease (ESLD) patients. Treatment with transplantation continues to advance with improvements in surgical techniques and in immunosuppressive therapy. Consequently, in the past few years, the early post‐OLT morbidity and mortality rate has significantly decreased. Today, the major focus, in the context of liver transplant recipients (LTRs), as well as in the context of other solid organ transplantations, has shifted to the care and management of long‐term complications. Long‐term complications in LTRs are mainly related to the detrimental effects of immunosuppression, especially the development of metabolic syndrome and its individual components, as well as the development of cardiovascular diseases and chronic kidney disease. Additionally, there is the risk of recurrence of primary disease. Finally, due to the improvement of surgical techniques, immunosuppressive medications, and the aging of the population, solid organ transplant recipients are becoming a growing population that is also facing some malignancy. Solid organ recipients are at almost three to 10 times higher risk of tumor development than non‐transplant patients because of the long‐standing immunosuppressed state. In the next few years, we are expecting groundbreaking changes in the context of OLT [1, 2, 3, 4, 5, 6].

During the last 10 years, immune checkpoint inhibitors (ICIs) have revolutionized the management of various cancer types and have become a standard of care for many patients with malignant diseases [1]. According to data, almost half of newly diagnosed patients with cancer are eligible for some of the approved ICIs [1]. The introduction of immunotherapy for cancer management, dominantly anti‐programmed death 1 (PD1)/PD1 ligand (PDL1) and anticytotoxic T‐lymphocyte‐associated protein 4 (CTLA‐4) medications, has raised concerns about its use in the context of solid organ transplantation. The main mechanism of action of ICIs is to block the negative regulation of effector T‐lymphocytes, which relates to the potentiation of anti‐tumor immune response in cancer patients. On the contrary, the same co‐inhibitory pathways that this medication suppresses are taking a role in maintaining organ tolerance [1, 7, 8]. Thus, clinical studies that have investigated ICIs excluded patients with a history of solid organ transplantation due to concerns of disrupting immune tolerance and consequently causing graft rejection [7, 8]. Also, the attention of the new studies is focused on the factors that influence the action of immunotherapy. Namely, the data indicate that tumor tissue can secrete bioactive factors called tumor‐derived supernatants (TDS), which can influence the response of the immune system in response to the tumor. Therefore, it is expected that a better understanding of TDS will contribute to a more adequate treatment of tumors [9].

Also, in addition to immunotherapy, research has recently been conducted on the combination of sorafenib and natural killer cells. Since both components are important in the treatment of HCC, it is assumed that their combination will lead to a synergistic effect and a better therapeutic response [10].

The use of ICIs in the context of solid organ transplantation raises two potential problems. One concern is that ICIs may cause severe, life‐threatening graft rejection [7, 8], and the second is that immunosuppressive drugs may reduce the efficiency of immunotherapy [5, 6, 7, 8, 11, 12, 13, 14]. According to data, the experience on the use of ICIs in the post‐LT setting is limited to case reports, case series, and small retrospective studies. Results of this data are variable with respect to anti‐tumor efficacy, as well as due to allograft rejection rates [1, 2, 3, 4, 5, 6, 7, 8, 11, 12, 13, 14, 15].

Nowadays, the efficiency of immunotherapy in non‐transplant patients and the availability of ICIs for cancer treatment is calling clinicians to estimate the potential benefits of ICIs in cancer management in solid organ recipients against the risk of graft rejection and potentially fatal outcomes [5, 6, 7, 8, 11, 12, 13, 14].

According to our data, there have been few case reports and several small retrospective studies investigating the use of ICIs in liver transplant recipients mainly with hepatocellular carcinoma (HCC) and skin cancer [7, 8, 11, 12, 13, 14, 15, 16, 17, 18], but there is no report regarding the use of atezolizumab for the treatment of metastatic breast cancer in liver transplant recipients.

## 2 Case History/Examination

In May 2019, a 61‐year‐old woman with a history of cryptogenic end‐stage liver disease underwent orthotopic liver transplantation. The liver graft was ABO type A and was from a young female patient, 24 years old. She was admitted to a hospital with a subarachnoid hemorrhage, and she suffered brain death. Immunosuppression after transplantation consisted of prednisone for 6 months on a tapering scheme, mycophenolate mofetil (MMF) 1 to 2 g/d for 12 months, and tacrolimus dosage titrated to targeted C0 concentration 4–8 ng/mL 1 month after transplantation. The patient did not experience early posttransplant complications. One year after the transplantation, she developed posttransplant diabetes mellitus and arterial hypertension. Later, in December 2020, in terms of posttransplant screening for malignancies, the suspected lump in the left breast was detected by mammography. She underwent an ultrasound‐guided core needle biopsies (CNB) of a 40 mm large solid mass. Histopathological examination revealed invasive ductal breast carcinoma, immunohistochemically triple‐negative, with Ki67 of 65% and PD‐L1‐expression > 1%. Ipsilateral axillary lymph nodes were not affected. According to the TNM classification she was characterized clinically T2N0. Prior to the initiation of neoadjuvant chemotherapy (NAC), staging scans revealed no distant metastasis. Table 1 shows the previously mentioned clinical findings.

**TABLE 1:** Initial clinical findings.

HISTOPATOLOGY AND IMMUNOHISTOCHEMISTRY		
Ki67	PD‐L1	Immunohistochemistry
65%	> 1%	Triple‐negative breast cancer
Diagnosis	Invasive ductal breast carcinoma	
TNM	T2N0	
ULTRASOUND		
40 mm large solid mass		

She was administered a dose‐dense AC protocol consisting of doxorubicin and cyclophosphamide followed by weekly paclitaxel (in a dose of 150 mg), which resulted in a partial tumor response verified by breast MRI.

After that she underwent partial mastectomy and sentinel lymph node biopsy. The final histopathological examination showed 1 cm residual invasive carcinoma and one positive sentinel lymph node. Post‐surgically, capecitabine treatment was initiated, and after 6 months of adjuvant chemotherapy, conformal photon radiotherapy of the left breast and regional lymph nodes with 50 Gy in 25 fractions was proceeded.

## 3 Methods/Differential Diagnosis, Investigations, and Treatment

**FIGURE 1:**
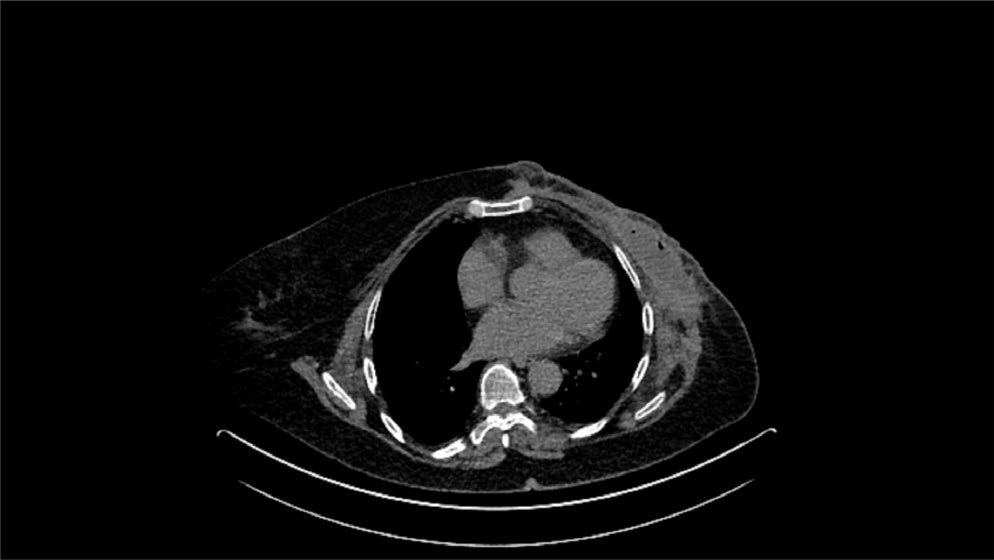
Thoracic CT scan in May 2022 reveals metastases in the left chest wall.

Two months after the radiation was completed, she presented with the lump in the left chest wall. Biopsy confirmed triple‐negative recurrence of the disease with metaplastic differentiation. Thoracic CT scan revealed metastases in the chest wall and spine, which were also confirmed with PET CT (Figure 1).

**FIGURE 2:**
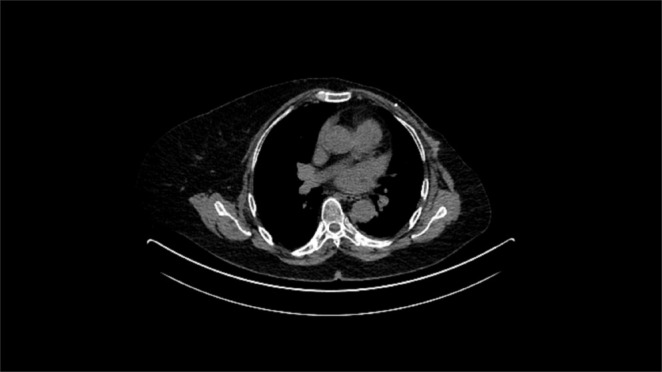
Last control CT evaluation in May 2024 shows no signs of disease.

Further steps included treatment with a combination of immunotherapy and chemotherapy—atezolizumab and nab‐paclitaxel. Prior to the introduction of immunotherapy the patient had a mild cholestatic lesion of the liver (ALP 142 IU/mL, GGT 96 IU/mL). In addition to chemoimmunotherapy, the patient continued her current immunosuppressive therapy and weekly controls of the tacrolimus blood concentration and liver enzymes. After 1 month, a slight increase was verified in cholestatic liver enzymes (ALP 196 IU/mL, GGT 114 IU/mL). Furthermore, abdominal ultrasound and color doppler of the transplanted liver were performed; apart from the hyperechoic liver in terms of liver steatosis, findings were normal. Magnetic resonance cholangiopancreatography showed no biliary obstruction. Blood tests showed that the level of tacrolimus was 3.6 ng/mL, which led to the increase of tacrolimus dosage from 1 + 1 to 1.5 + 1.5 mg per day. The systemic therapy was continued with further monitoring of tacrolimus blood concentrations and liver enzymes. Regarding the patient's renal function, during ICIs treatment, it remained stable. Onwards, findings of follow‐up imaging showed regression of bone metastases. After the end of oncological therapy, the function of the liver transplant was stable, and further monitoring by a hepatologist and oncologist was continued. A recent follow‐up in May 2024 suggested that the patient was in fair condition and no clinical or radiological signs of recurrence have been found (Figure 2).

Table 2 shows the values of liver enzymes and tacrolimus concentrations during the administration of chemoimmunotherapy.

**TABLE 2:** Liver enzyme levels and tacrolimus concentrations during the ICU treatment.

	Normal values	0 Months	1st month	3rd month	6th month	9th month	12th month	18th month
AST (IU/mL)	11–38	22	21	28	24	22	27	25
ALT (IU/mL)	12–48	26	24	30	28	29	26	24
GGT (IU/mL)	11–55	96	114	99	92	90	88	82
ALP (IU/mL)	60–142	142	196	182	183	182	185	180
Bilirubin (mmol/L)	3–20	16	18	21	19	24	18	21
Tacrolimus ng/mL^a^		4.5	3.6	4.6	4.5	4.8	4.6	4.5

^a^ Normal values of tacrolimus are related to the time of transplantation.

## 4 Discussion

Parallel with the aging of the population, the increased need for liver transplantation treatment, the population of LTRs that will develop some form of cancer is growing. Development of cancer in that population is directly related to the long‐standing use of immunosuppressive therapy [1, 2, 3, 4, 5, 6, 14, 15, 16, 17, 18, 19]. During the ICIs clinical trials, the solid organ recipients were not included since ICIs have an important role in self‐ as well as in allograft tolerance. According to data, the risk of acute allograft rejection is ranged between 10% to 65% [7].

As a result, our knowledge regarding the use, safety, and efficacy of ICIs in solid organ recipients remains limited. In contrast, the influence of immunosuppressive medications on the efficacy of ICI is also understudied [8]. The first recorded case report of ICIs use in a kidney transplant recipient was published in 2014, and since then, off‐label data on their use in patients with organ transplants have been increasing [20].

Today, ICIs are the standard of care for many advanced malignancies; however, pre‐clinical data show that the same co‐inhibitory signals that ICIs suppress are involved in maintaining organ tolerance [8]. Therefore, ICIs may disrupt the equilibrium of immunological tolerance, consequently leading to acute rejection [8, 20, 21, 22, 23, 24]. These mechanisms are not fully understood. Some data suggest that proteins such as CTLA‐4, PD‐1, and PD‐L1 play an important role in graft tolerance. Protein CTLA‐4 has a crucial role in enhancing T‐regulatory activity. Conversely, it also decreases the activity of T‐helper cells, which relates to allograft/immune tolerance after transplantation. Furthermore, it has been shown that the PD‐1/PD‐L1 axis promotes inhibition of alloreactive T‐cell activation, while this axis promotes the development of regulatory T‐cells that are also associated with immune tolerance during the post‐LT period [20, 21, 22, 23, 24].

There are more than 30 studies, mostly case reports, case series, small retrospective studies, that evaluated around 60 LT patients who were treated with ICIs mainly in cases with recurrent hepatocellular carcinoma, melanoma, and skin cancer. The most used ICI medications were nivolumab and the combination of atezolizumab and bevacizumab, which was prescribed to patients with HCC recurrence after LT. The follow‐up period was from 7 to 10 months, and the acute rejection rate was up to 25% [4, 6, 14, 15, 17, 19, 22]. A recent meta‐analysis involved 28 LT patients that were given ICIs—25 patients from 16 publications, 3 patients from their institutional database [23]. According to the analysis, the rejection rate was 32%. The risk was associated with the time since transplantation, as LTRs who experienced an acute rejection crisis were administered ICIs earlier [23]. Like this meta‐analysis, some other data show a higher incidence of acute rejection if ICI is given soon after LT. One of the factors also connected to a higher incidence of rejection is the type of ICI that was administered—the rejection rate among LTRs treated with PD‐1 inhibitors was higher than that of LTRs who received anti‐CTLA‐4 therapy [24]. Authors assumed that LTRs with PD‐1/PD‐L1 positive histology appear to carry a higher risk of rejection. Thus, it has been proposed that liver biopsy could be performed before the administration of ICIs to predict the risk for rejection [24]. This approach could be a useful tool, but it needs to be confirmed in further analysis and studies. Regarding the response rate and the time frame between ICI therapy and LT, some data report that LTRs who responded well to ICI therapy had a longer time frame between the start of ICI treatment and LT compared to those who were non‐responders (6 vs. 3 years) [24]. Thus, it is possible that the immune system of LTRs with older and more immune‐tolerant allografts is more focused on the malignancy than on the transplanted liver [24].

According to data, there was no case report regarding the use of atezolizumab after liver transplantation in the indication of triple‐negative de novo breast cancer. Around 10%–20% of all breast cancer cases are of the triple‐negative type, which is characterized by aggressive clinical courses and early metastasis [25]. In our case, the maintenance of the patient's immunosuppressive therapy with tacrolimus and management with the anti–PD‐L1 inhibitor, atezolizumab, was associated with a rapid response and significant resolution of metastatic bone disease, with no long‐term evidence of allograft rejection. We observed a complete response after 6 cycles of therapy, with a sustained response 18 months after initiation. Treatment with anti‐PD‐L1 inhibitors can be administered to liver transplant recipients with breast metastatic disease but demands careful monitoring of liver function and immunosuppression exposure. Thus, our case emphasizes the need for a case‐by‐case approach in LTRs, considering that breast cancer is common both in the general population and among LTRs. Additionally, we now have a therapy (i.e., immunotherapy) with a good clinical response, which should be considered through a multidisciplinary approach for LTRs with malignancies.

## 5 Conclusion

Data regarding the use of ICI after liver transplantation is insufficient, and our knowledge regarding the efficiency and safety of ICI use in liver transplant recipients is limited. Although the risk of graft rejection in this population of patients is high, there is a good response to ICIs and increased survival in those liver transplant recipients that do not experience rejection. The use of ICIs following LT is more challenging; thus, a case‐by‐case‐based approach is needed in the context of ICI administration in this population of patients. Ultimately, a multidisciplinary approach should be a standard of care for each of these challenging patients, as it was in our patient. Finally, all risks and benefits, including the risk of fatal graft failure, must be extensively discussed with each liver transplant recipient. Further studies could focus on investigating PD‐L1 status of liver grafts to help clarify if graft PD‐L1 status predicts the risk of rejection.

## Author Contributions

**Iva Skocilic:** conceptualization, data curation, formal analysis, investigation, methodology, resources, software, supervision, validation, visualization, writing – original draft, writing – review and editing. **Alojzije Kosic:** conceptualization, data curation, investigation, resources, software, validation, visualization, writing – original draft, writing – review and editing. **Ivona Badovinac:** conceptualization, data curation, formal analysis, investigation, resources, software, supervision, validation, visualization, writing – original draft, writing – review and editing. **Tajana Filipec Kanizaj:** conceptualization, resources, software, supervision, validation, visualization, writing – original draft, writing – review and editing. **Renata Dobrila Dintinjana:** conceptualization, resources, software, supervision, validation, visualization, writing – original draft, writing – review and editing. **Maja Kolak:** resources, software, validation, visualization, writing – original draft, writing – review and editing. **Anamarija Bukovica:** resources, software, supervision, validation, visualization, writing – original draft, writing – review and editing. **Sanja Ropac:** resources, software, supervision, validation, visualization, writing – original draft, writing – review and editing. **Ivana Mikolasevic:** conceptualization, data curation, formal analysis, investigation, methodology, project administration, resources, supervision, validation, visualization, writing – original draft, writing – review and editing.

## Consent

Written informed consent was obtained from the patient to publish this report in accordance with the journal's patient consent policy.

## Conflicts of Interest

The authors declare no conflicts of interest.

## Data Availability

The data that support the findings of this study are available on request from the corresponding author. The data are not publicly available due to privacy or ethical restrictions.
